# Fucoidan Treatment Improves Diabetic Hyperglycemia and Dyslipidemia in Rodents: A Systematic Review and Meta-Analysis

**DOI:** 10.3390/nu18071155

**Published:** 2026-04-03

**Authors:** Kana Watanuki, Rin Akiyama, Shiita Watanabe, Eri Adachi, Masako Shimada

**Affiliations:** 1 Faculty of Nutritional Science, Sagami Women’s University, 2-1-1 Bunkyo, Minami-ku, Sagamihara 252-0383, Kanagawa, Japan; 2 Graduate School of Nutritional Science, Sagami Women’s University, 2-1-1 Bunkyo, Minami-ku, Sagamihara 252-0383, Kanagawa, Japan

**Keywords:** fucoidan, diabetic rodent models, hyperglyceridemia, dyslipidemia, meta-analysis

## Abstract

**Background/Objectives**: Fucoidan is a sulfated long-chain polysaccharide found mainly in sea cucumbers and brown algae. Studies suggest that fucoidan may play a role in treating various diseases, including metabolic syndrome and diabetes mellitus. The purpose of the current study was to investigate the effects of fucoidan isolated from brown algae on diabetic hyperglycemia and dyslipidemia. **Methods**: Two databases, PubMed and Embase, were searched to identify peer-reviewed articles written in English and published up to 30 June 2025. Studies reporting blood glucose and serum/plasma lipid levels of diabetic rodents treated with fucoidan or vehicle were included in the meta-analysis. **Results**: Forty-seven studies reported blood glucose levels. The pooled effect size for blood glucose levels was −2.26 (95% CI: −2.78 to −1.75), with substantial heterogeneity. Subsequent analyses showed that diabetic dyslipidemia was markedly improved in the fucoidan-treated group compared with the control. **Conclusions**: Fucoidan treatment could improve hyperglycemia and dyslipidemia in diabetic rodents.

## 1. Introduction

Diabetes mellitus (DM) is a chronic endocrine disorder characterized by sustained elevated blood glucose levels; this condition often starts with the body’s resistance to insulin action and can develop into the inability to produce sufficient insulin [1]. The prevalence of DM continues to increase worldwide. The International Diabetes Federation estimated that 589 million people between 20 and 79 years of age were living with diabetes in 2024 and predicted that the number would increase to 853 million by 2050. Diabetic dyslipidemia, characterized by hypertriglyceridemia, hypercholesterolemia, and decreased high-density lipoprotein cholesterol (HDL-C) levels, has been recognized as a crucial risk factor for cerebral and cardiovascular disorder development and death among elderly individuals [1].

Brown algae encompass diverse species of seaweed, including *Sargassum fusiforme*, *Saccharina japonica* (formerly named *Laminaria japonica),* and *Undaria pinnatifida*, and contain more sulfated polysaccharides than green and red algae [2]. Among these polysaccharides are fucoidans: fucose-containing polysaccharides composed of L-fucose residues bound via α-(1→2), α-(1→3), and/or α-(1→4) glycosidic bonds, with trace amounts of mannose, xylose, and galactose [3]. Fucoidans play a particularly crucial role as dietary fiber and have been recognized for their numerous health benefits against some types of cancer, obesity, and DM [4,5]. Evidence from in vitro and in vivo studies suggests the positive influences of fucoidan on glucose and lipid metabolism [6]. However, relatively few clinical studies on fucoidan’s role in humans have been reported to date [7,8,9,10]. Therefore, this research aimed to investigate the effects of fucoidan on diabetic hyperglycemia and dyslipidemia by conducting a meta-analysis of the available evidence from DM mouse and rat models.

## 2. Materials and Methods

### 2.1. Date Sources and Search Strategies

The electronic databases PubMed and Embase were searched using the terms (“fucoidan” and (“diabetes mellitus” or “insulin resistance” or “metabolic syndrome” or “syndrome X”) to identify research published up to 30 June 2025. The reference lists of the identified articles were also examined to ensure that all related reports would be included.

### 2.2. Inclusion and Exclusion Criteria

**Figure 1:**
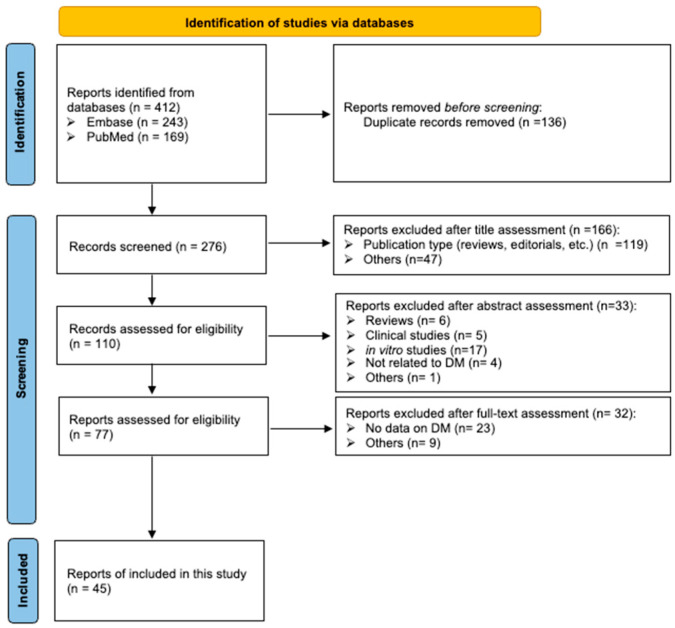
Flow diagram of literature search.

The inclusion criteria were (i) peer-reviewed articles written entirely in English; (ii) treatment of mouse/rat DM models with fucoidan or vehicle for 5 days or longer; and (iii) measurement of the blood glucose, triglyceride (TG), total cholesterol (TC), or HDL-C levels of the DM animals at the end of the fucoidan or vehicle treatment. Articles were excluded if they were duplicates, editorials, reviews, letters, or meeting abstracts; were not written in English; or did not report data on blood glucose or lipid levels. Unpublished studies were also excluded. The systematic review was conducted in accordance with PRISMA 2000 guidelines [11]. The templates for the flow diagram and checklist were downloaded from https://www.prisma-statement.org/prisma-2020 (accessed on 18 January 2026) (Figure 1, Table S1).

### 2.3. Data Extraction and Quality Assessment

The titles, abstracts, and main texts of the identified articles were independently screened by two authors for potentially eligible studies. Relevant data were then extracted using a predesigned data extraction form, including the fucoidan dose, molecular weight, treatment duration, blood glucose and lipid (TG, TC, and HDL-C) levels, age or body weight, sex, and types of rodents and DM models. The quality of each study was evaluated by the Cochrane’s “Risk of Bias” Tool [12,13]. Two authors again independently assessed the risk of bias of each quality variable and judged it as “low”, “medium”, “high”, “not clear”, or “not applicable (N/A)” based on the description of the included articles. Any disagreements at any phase were resolved by intensive discussion until a consensus was reached.

### 2.4. Data Synthesis and Analysis

Continuous variables are shown as means ± standard deviations (SDs). When studies reported variables as means ± standard errors of means (SEs), the corresponding SDs were calculated using the SEs and sample sizes. If glucose levels were provided in mmol/L, they were converted to mg/dL, as described previously [14]. If serum/plasma TG, TC, and HDL-C levels were expressed in mmol/L, they were converted to mg/dL, as described previously [14]. As a rule, when studies reported multiple measures of blood glucose and serum/plasma lipid levels, the values after the longest period of fucoidan treatment and at the highest treatment dose were used. Animals treated with either streptozotocin (STZ) or alloxan were assigned to the chemical-induced DM model group, even if they were fed a high-calorie diet.

Hedges’ g transformation was used to calculate related statistics, including the summary effect size, variance, and 95% confidence intervals (CIs) in each study, as described previously [14]. The meta-analysis was performed with a random-effects model. Heterogeneity was calculated using the *I*^2^ statistics, where a value over 75% was interpreted as evidence of high heterogeneity [15].

Subgroup analyses were conducted on the basis of the DM model type (chemical-induced by STZ or alloxan; diet-induced by high-fat diet (HFD) or high-fat and high-sugar diet (HFSD); or genetically altered non-obese diabetic (NOD) and db/db mice or Goto Kakizaki (GK) rats), sex (males or females), the molecular weight of fucoidan (low-molecular-weight fucoidan (LMWF): ≤15 kDa; high-MW fucoidan (HMWF): >15 kDa), and the family of brown algae from which fucoidan was extracted. Meta-regression analyses were performed regarding treatment dose and period to test their impact on the outcome estimates. Sensitivity was analyzed using a leave-one-out strategy to assess the influence of each study on the outcome estimates. Publication bias was evaluated by funnel plots together with Duval and Tweedie’s Trim-and-Fill analysis (random-effects model). Statistical analysis was conducted using STATA/BE19.0 (Stata Corp, College Station, TX, USA) and Comprehensive Meta-Analysis 4.0 (Biostat Inc., Englewood, NJ, USA) software. A *p*-value less than 0.05 was judged as statistically significant.

## 3. Results

### 3.1. Search Results

The flow diagram of the literature search is shown in Figure 1. The search returned 412 reports in total (243 in Embase and 169 in PubMed). After duplicates were removed, 276 reports proceeded to title assessment. Abstracts were screened for 110 reports, of which 77 underwent a subsequent full-text screening. Thirty-two reports were further excluded because they did not report blood glucose or lipid levels of DM rodent models. Thus, 47 studies described in 45 articles were included in this meta-analysis.

### 3.2. Study Characteristics and Quality Assessment

Table 1 summarizes the baseline characteristics of each included study. The included studies were published between 2012 and 2025, with sample sizes ranging from 10 to 34. Animals were treated with fucoidan isolated from various brown algae, including *Saccharia japonica*, *Sargassum fusiforme*, and *Undaria pinnatifida*. Chemically induced DM rodents injected with STZ or alloxan were used in 21 studies (rats in 15, mice in six studies), genetically altered DM rodents (db/db or NOD mice, GK rats) in 14 studies (rats in four, mice in 10 studies), and diet-induced obese DM rodents in 12 studies (rats in two, mice in 10 studies). Forty-two studies used males, three used females, and two studies did not specify the sex of experimental animals. The quality assessment of each study is recorded in Table S2. The study quality was generally fair, with the risk of bias judged to be low to medium.

### 3.3. Effect of Fucoidan on Blood Glucose Levels in DM Mouse or Rat Models

#### 3.3.1. Forest Plot Analysis and Assessment of Publication Bias

**Figure 2:**
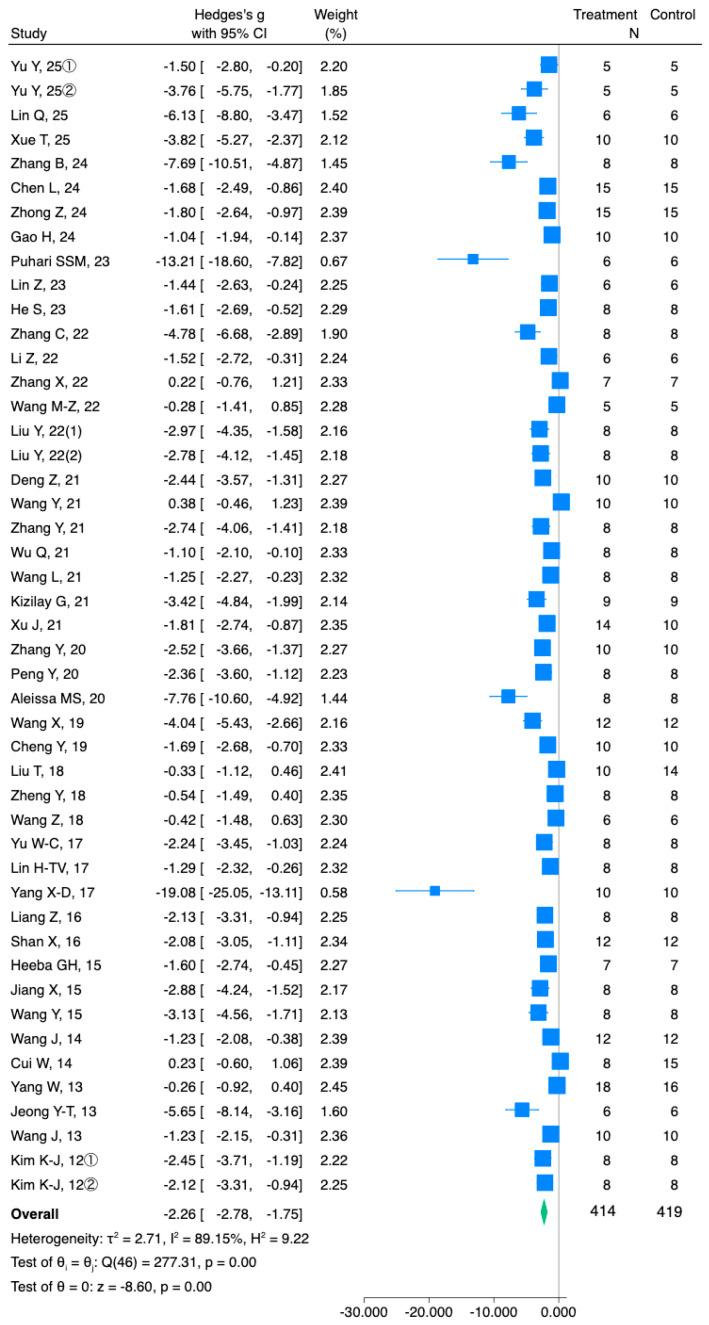
Meta-analysis of Hedges’ g of blood glucose levels of DM rodents treated by fucoidan or vehicle. Summary estimates were assessed using a random-effects model. CI, confidence interval [16,17,18,19,20,21,22,23,24,25,26,27,28,29,30,31,32,33,34,35,36,37,38,39,40,41,42,43,44,45,46,47,48,49,50,51,52,53,54,55,56,57,58,59,60].

Forty-seven studies reported blood glucose levels; they included 414 and 419 diabetic animals orally administered either fucoidan or vehicle, respectively. Thirty-nine studies reported that administration of fucoidan markedly reduced blood glucose levels of DM rodents; eight studies detected no significant effects of fucoidan. The random-effects model used for the meta-analysis revealed that fucoidan reduced blood glucose levels of DM rodents compared with vehicle (Hedges’ g = −2.26, 95% CI −2.78 to −1.75; *I*^2^ = 89.15%, *p* = 0.00) (Figure 2). The results’ stability was tested using sensitivity analysis to determine the influence of each study on the overall result. No re-pooled summary estimates were significantly altered by the removal of an individual study compared with the primary values; the effect sizes ranged from −2.31 (95% CI, −2.83 to −1.80) to −2.11 (95% CI, −2.57 to −1.66).

**Figure 3:**
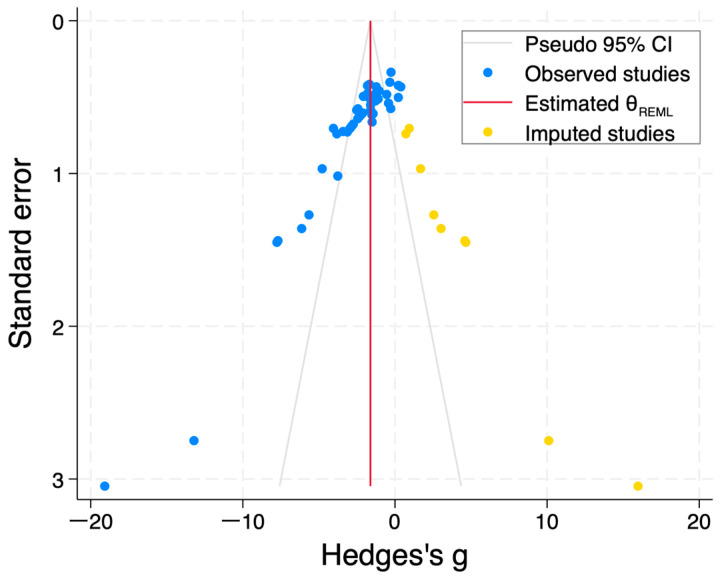
Funnel plots of standard error (SE) by Hedges’ g of blood glucose levels of DM rodents administered either fucoidan or vehicle. Blue and Yellow dots indicate original and imputed summary estimates, respectively.

The above results suggest that administration of fucoidan reduces blood glucose levels compared with vehicle in DM rodents, with high heterogeneity; thus, publication bias was evaluated. Duval and Tweedie’s Trim-and-Fill analysis identified nine imputed studies (orange dots) for blood glucose levels, and the adjusted g (95% CIs) was −1.62 (−2.45 to −0.78) (Figure 3). Thus, publication bias was detected in studies examining blood glucose levels of DM rodents; however, the above finding that fucoidan treatment could beneficially affect blood glucose levels of DM rodents held after Trim-and-Fill adjustment.

#### 3.3.2. Subgroup and Meta-Regression Analyses

To identify categorical covariates that influenced the between-study heterogeneity of the meta-analysis, subgroup analysis was conducted based on the types of DM models and rodent, the MW of fucoidan, and the family of brown algae from which fucoidan had been extracted. The analysis revealed no significant covariates (Table S3). Meta-regression analysis was then performed to test whether the daily dose, treatment period, or sulfation levels of fucoidan were potential moderators. The results showed that none of these factors significantly influenced the variance. Ultimately, the cause of high heterogeneity in blood glucose levels across the 47 pooled studies was not determined. Therefore, although our meta-analysis suggests that fucoidan may contribute to reducing blood glucose levels in DM rodents, the evidence level of this finding is considered low due to high heterogeneity.

#### 3.3.3. Forest Plot and Publication Bias in Chemical-Induced DM Rodents

Because the subgroup analysis was somewhat suggestive of a group difference among DM rodent models, analyses were performed separately in chemical- and diet-induced and genetically altered DM models (Table S3). A meta-analysis of 21 studies revealed that fucoidan treatment decreased blood glucose levels of chemical-induced DM rodents vs. vehicle (Hedges’ g = −3.25, 95% CI −4.66 to −1.84; *I*^2^ = 96.54%, *p* < 0.001) (Table S3). However, the Trim-and-Fill analysis found four imputed studies. Hedges’ g (95% CIs) after adjustment was −1.934 (−4.09 to 0.22). Thus, fucoidan administration did not alter blood glucose levels in chemical-induced DM rodents.

#### 3.3.4. Forest Plot, Publication Bias, and Subgroup and Meta-Regression Analyses in Diet-Induced DM Rodents

**Figure 4:**
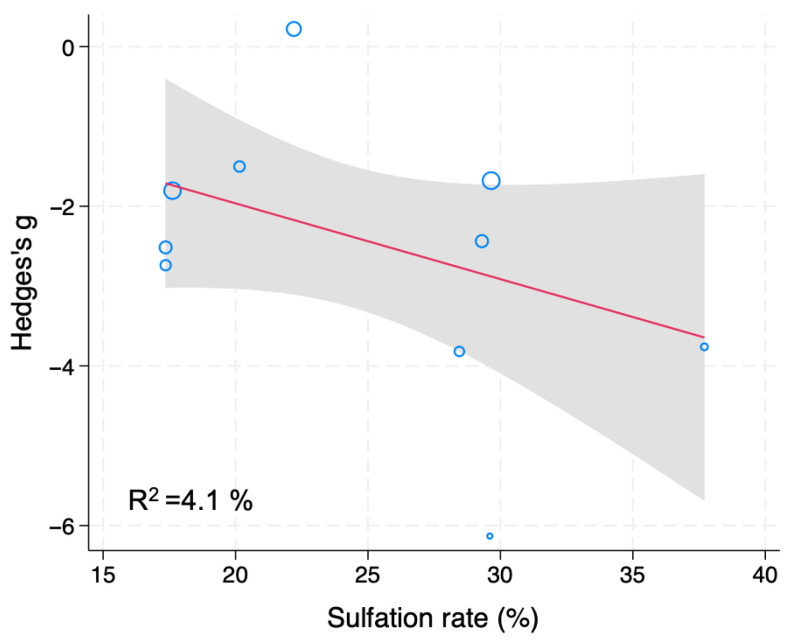
Meta-regression analysis of Hedges’ g for blood glucose levels of diet-induced DM rodents treated with fucoidan or vehicle. Blue open circles, a red line and shaded area indicate studies, linear prediction, and 95%CI, respectively.

Twelve studies showed that fucoidan significantly lowered blood glucose levels of diet-induced DM animals (g = −2.18, 95% CI −2.92 to −1.44, *I*^2^ = 78.50%, *p* < 0.001) (Table S3). Trim-and-Fill analysis detected no publication bias. Subgroup and meta-regression analyses were then conducted to identify influencing factors for blood glucose levels of diet-induced DM rodents. The sulfation rate of fucoidan (R^2^ = 4.1%) was found to be a covariate (Figure 4). In sum, fucoidan administration reduces blood glucose levels of diet-induced DM rodents; the sulfation rate of fucoidan might, at least partially, explain the high heterogeneity.

#### 3.3.5. Forest Plot, Publication Bias, and Subgroup and Meta-Regression Analyses in Genetically Altered DM Rodents

Administration of fucoidan reduced blood glucose levels of genetically altered DM model animals (g = −1.56, 95% CI −2.19 to −0.93, *I*^2^ = 78.44%, *p* < 0.001) (Table S3). Trim-and-Fill analyses showed no publication bias. Subgroup and meta-regression analyses were then conducted. The family of brown algae was identified as a significant confounding factor (*p* < 0.001) (Table 2). Thus, fucoidan administration reduces blood glucose levels of genetically altered DM rodents, and the family of brown algae used may be a covariate in its effect. However, some families of brown algae were examined in only a few studies (*n* < 4); thus, more studies are needed for further evaluation.

Collectively, the results show that fucoidan administration may exert a beneficial impact on blood glucose levels in diet-induced and genetically altered DM rodents. The sulfation rate of fucoidan and the family of brown algae could be, at least partially, responsible for the heterogeneity in diet-induced and genetically altered DM rodent models, respectively.

### 3.4. Effect of Fucoidan on Serum/Plasma Lipid Levels of DM Rodent Models

#### 3.4.1. Forest Plot Analysis and Publication Bias Assessment

**Figure 5:**
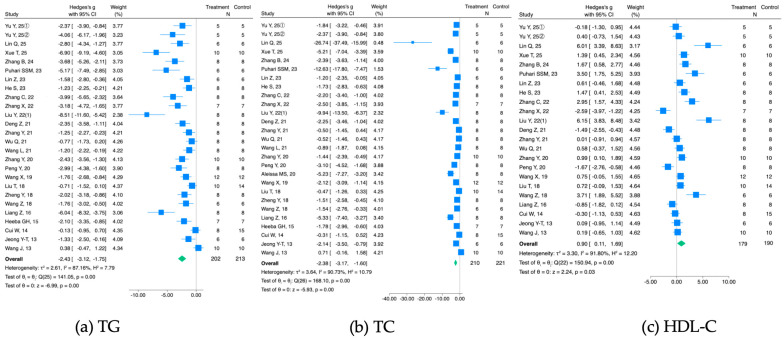
Meta-analysis of Hedges’ g of serum/plasma TG (**a**), TC (**b**), and HDL-C (**c**) levels of DM mice/rats with and without fucoidan treatment [16,17,18,19,23,24,25,26,28,30,32,34,35,36,39,40,41,42,44,45,46,50,52,56,58,59].

Twenty-six studies involving 202 DM rodents treated with fucoidan and 213 DM control rodents reported the serum/plasma TG levels; 27 studies (210 fucoidan-treated DM rodents and 221 DM controls) reported the TC levels; and 23 studies (179 fucoidan-treated DM rodents and 190 DM controls) reported the HDL-C levels. The meta-analyses indicate that fucoidan administration significantly reduces TG (g = −2.43, 95% CI −3.12 to −1.75; *I*^2^ = 87.16%, *p* = 0.00) and TC (g = −2.38, 95% CI −3.17 to −1.60; *I*^2^ = 90.73%, *p* = 0.00) levels compared with vehicle in DM rodents, while it increases serum/plasma HDL-C levels (g = 0.90, 95% CI 0.11 to 1.69; *I*^2^ = 91.80%, *p* = 0.00) (Figure 5). Next, the results’ stability was tested, as described above. The re-pooled summary estimates ranged from −2.54 (95% CI, −3.20 to −1.88) to −2.25 (95% CI, −2.86 to −1.63) for TG, from −2.48 (95% CI, −3.30 to −1.67) to −2.08 (−2.71, −1.44) for TC, and from 0.69 (−0.01, 1.38) to 1.04 (0.28, 1.79) for HDL-C levels.

**Figure 6:**
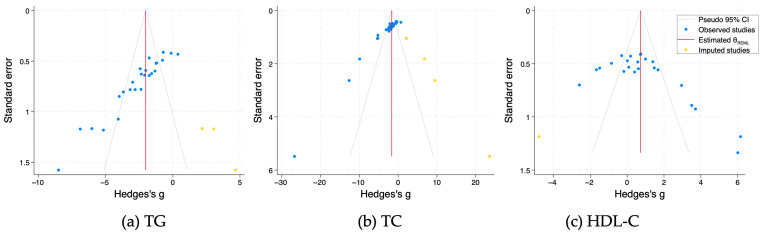
Funnel plots of SE by Hedges’ g of serum/plasma TG (**a**), TC (**b**), and HDL−C (**c**) levels in DM rodents treated with fucoidan or vehicle. Blue and yellow dots indicate the original and imputed summary estimates.

Publication bias for serum/plasma TG, TC, and HDL-C levels of DM animals was then assessed. Duval and Tweedie’s Trim-and-Fill analyses detected three, five, and one imputed study (orange dots) for TG, TC, and HDL–C levels, respectively (Figure 6). The adjusted g (95% CIs) was −2.02 (−2.88 to −1.71) for TG, −1.66 (−3.09 to −0.22) for TC, and 0.73 (−0.14 to 1.59) for HDL-C levels. Taken together, the results indicate a publication bias in studies of DM rodents, but the main finding that fucoidan treatment beneficially affected serum/plasma TG and TC levels of DM rodents still held after the adjustment.

#### 3.4.2. Subgroup and Meta-Regression Analyses

**Figure 7:**
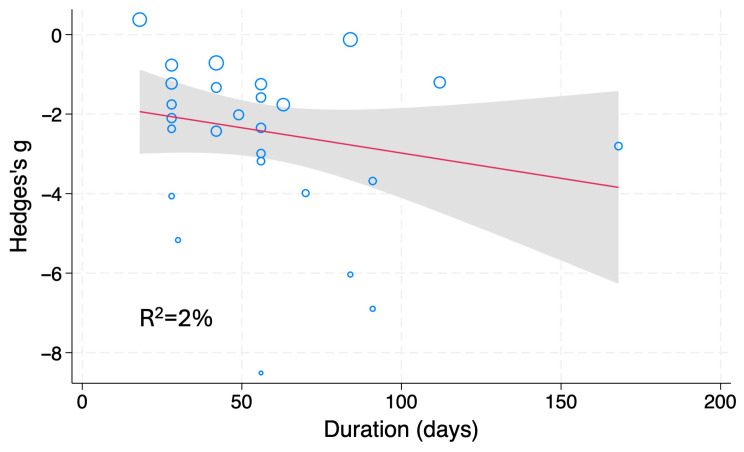
Meta-regression analysis of Hedges’ g for serum/plasma TG levels of DM rodents treated with fucoidan or vehicle. Blue open circles, a red line and shaded area indicate studies, linear prediction, and 95%CI, respectively.

Subgroup and meta-regression analyses were conducted, as previously described. The results showed that the type of DM model and the treatment period (R^2^ = 2%) were significant factors for TG; the types of DM model and rodent and the MW of fucoidan were significant factors for TC levels (Table 3, Figure 7). Thus, the high heterogeneity in hypertriglyceridemia and hypercholesterolemia among the pooled studies is due, in part, to the type of DM rodents.

#### 3.4.3. Forest Plot, Publication Bias, and Subgroup and Meta-Regression Analyses in Chemical-Induced DM Rodents

Analyses were then performed separately in chemical- and diet-induced and genetically altered DM models. Ten and eleven studies showed that chemically induced DM rodents treated with fucoidan exhibited significant decreases in serum/plasma TG and TC levels, respectively (TG, g = −3.14, 95% CI −4.70 to −1.57, *I*^2^ = 92.84%, *p* = 0.00; TC, g = −3.57, 95% CI −5.56 to −1.59, *I*^2^ = 95.82%, *p* = 0.00) (Table 3). The Trim-and-Fill analyses found no imputed studies for TG and one imputed study for TC levels. Hedges’ g (95% CIs) after the adjustment for TC was −3.03 (−5.36 to −0.70).

**Figure 8:**
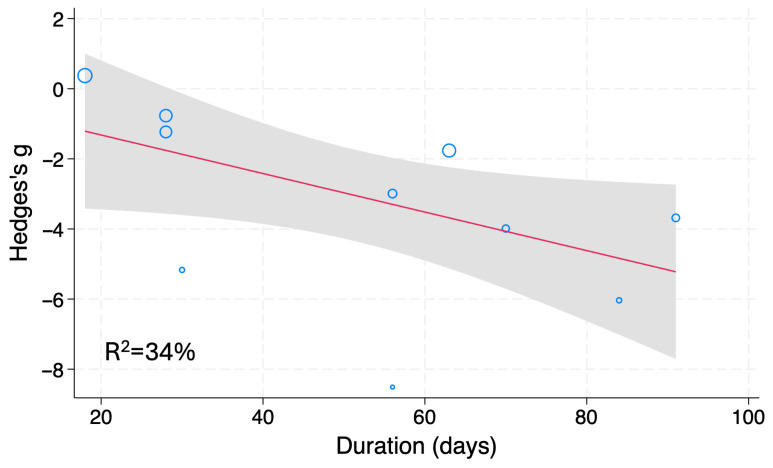
Meta-regression analysis of Hedges’ g of serum/plasma TG levels of chemical-induced DM animals treated with fucoidan or vehicle. Blue open circles, a red line and shaded area indicate studies, linear prediction, and 95%CI, respectively.

Subgroup and meta-regression analyses were conducted to identify factors that significantly influence the serum/plasma TG and TC levels of chemical-induced DM models. The MW of fucoidan and the treatment period (R^2^ = 34%) were found to be covariates for TG in chemical-induced DM animals (Table 4, Figure 8). However, the number of studies that used LMWF was low (*n* = 2); thus, more studies are needed for further evaluation. No significant factors were identified as being responsible for the heterogeneity of TC in this animal model.

#### 3.4.4. Forest Plot, Publication Bias, and Subgroup and Meta-Regression Analyses in Diet-Induced DM Rodents

Ten studies showed that fucoidan significantly lowed serum/plasma TG and TC levels of diet-induced DM models (TG, g = −2.63, 95% CI −3.46 to −1.80, *I*^2^ = 72.61%, *p* = 0.001; TC, g = −2.12, 95% CI −2.98 to −1.26, *I*^2^ = 76.14%, *p* = 0.02) (Table 3). Trim-and-Fill analyses detected one imputed study each for TG and TC levels. The values of adjusted Hedge’ g (95% CIs) were −2.34 (−3.48 to −1.20) for TG and −2.07 (−6.81 to 2.68) for TC. Therefore, fucoidan significantly reduces serum/plasma levels of TG but not TC in diet-induced DM animals.

**Figure 9:**
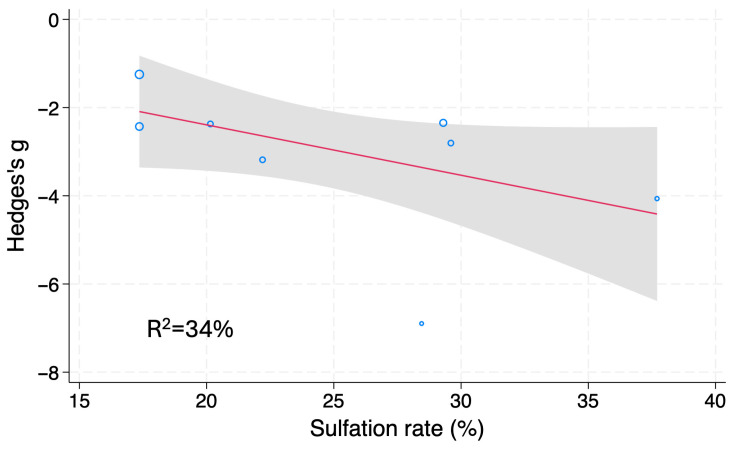
Meta-regression analysis of Hedges’ g of serum/plasma TG levels of diet-induced DM rodents treated by fucoidan or vehicle. Blue open circles, a red line and shaded area indicate studies, linear prediction, and 95%CI, respectively.

Subgroup and meta-regression analyses were performed to identify influencing factors for serum/plasma TG levels of diet-induced DM rodents. The family of brown algae and the sulfation rate of fucoidan (R^2^ = 34%) were found to be significant covariates (Table 5, Figure 9). Collectively, the results indicate that fucoidan administration reduces serum/plasma TG levels of diet-induced DM rodents; the family of brown algae from which fucoidan is extracted and the sulfation rate of fucoidan may influence the overall outcome. However, the number of studies using fucoidan harvested from the families Fucaceae and Sargassaceae was low (*n* = 2 each); therefore, more studies are required for further assessment.

#### 3.4.5. Forest Plot, Publication Bias, and Subgroup and Meta-Regression Analyses in Genetic DM Rodents

Six studies showed that fucoidan significantly lowered serum/plasma TG and TC levels in genetic DM models (TG, g = −1.15, 95% CI −1.77 to −0.54, *I*^2^ = 51.94%, *p* = 0.001; TC, g = −1.07, 95% CI −1.63 to −0.50, *I*^2^ = 44.38%, *p* = 0.00) (Table 3). Duval and Tweedie’s Trim-and-Fill analyses detected no imputed studies for TG or TC levels. Moreover, between-study heterogeneity was lower than 55%. Therefore, no additional subgroup and meta-regression analyses were conducted. In sum, fucoidan treatment showed a beneficial effect on serum/plasma TG and TC levels of genetically altered DM models.

## 4. Discussion

### 4.1. Main Findings

Table 6 summarizes the effect of fucoidan administration on the levels of blood glucose, TG, TC, and HDL-C of the three types of DM models. This meta-analysis revealed evidence that administration of fucoidan to DM rodents significantly reduces their blood glucose and serum/plasma TG and TC levels compared with vehicle (Figure 5). These results were, at least partially, affected by the type of DM rodent model (Table 3). Furthermore, in diet-induced and genetic DM rodents, blood glucose levels could be affected by the sulfation rate of fucoidan and the family of brown algae from which it originated, respectively. Serum/plasma TG levels in chemical- and diet-induced rodents might be influenced by the fucoidan treatment period and fucoidan sulfation rate, respectively.

The overall assessment of the quality of evidence of the meta-analysis is summarized in Table 7 [61]. The certainty of the evidence regarding fucoidan’s effect on blood glucose levels was judged as “very low” because the analysis showed very high heterogeneity, and no definitive confounding factors were identified in the primary analysis. Subsequently, we showed that fucoidan’s beneficial effect on blood glucose levels was limited to diet-induced and genetically altered DM rodents and identified confounding factors that might at least partially explain the heterogeneity. While the certainty of evidence on serum/plasma TG and TC levels was “moderate”, fucoidan administration was associated with a significant reduction in their levels, particularly in genetic DM rodent models.

### 4.2. Interpretation

Fucoidans are water-soluble fucose-containing sulfated polysaccharides with a relatively complex structure. They typically consist of alternating α(1→3)- and α(1→4)-fucoside bonds, with a variety of monosaccharides, including galactose, mannose, rhamnose, xylose, and glucuronic acid, attached to the main chain [3,62]. However, the basic structure of fucoidans varies depending on the species of brown algae, the purification method, and certain chemical reactions, including desulfation and methylation [6]. The MW of fucoidans generally ranges from 100 to 1600 kDa [63]. The low bioactivity and bioavailability of fucoidans have been attributed to their high MW and the structural complexity and diversity [64]. Thus, fucoidans with a low molecular weight and/or a higher sulfation rate have recently gained considerable attention for pharmaceutical applications because a decrease in the MW of fucoidan, with or without a higher sulfation rate, was shown to significantly improve both its absorption and bioavailability in animal models [16,65]. Interestingly, in this meta-analysis, the MW of fucoidan was found to be a covariate for serum/plasma TC levels of DM rodents (Table 3) and to affect serum/plasma TG levels of chemical-mediated DM rodents (Table 4). Moreover, the sulfation rate of fucoidan was found to be a significant factor influencing blood glucose and serum/plasma TG levels of diet-induced DM rodents (Figure 4, R^2^ = 4.1%; Figure 9, R^2^ = 34%). Therefore, more future studies are required to further define the roles of MW and sulfation rate of fucoidan in diabetic hyperglycemia and dyslipidemia.

The type of DM models was identified as a significant covariate influencing serum/plasma TG and TC levels of fucoidan-treated DM rodents in this study. STZ- or alloxan-induced DM rodents and NOD mice are often referred to as insulin-dependent DM animals, unable to produce and secrete sufficient insulin from pancreatic β-cells [66,67]. HFD or HFSD-induced and genetically induced DM models—db/db and GK rats—are considered models of Type 2 DM (T2DM), marked by overweight and insulin resistance [68]. DM severity and progression may differ among those animal models. Therefore, it is plausible that the effect of fucoidan treatment varies among DM patients depending on the etiology and stage of the disease and their daily lifestyle.

#### 4.2.1. Strength and Limitations

The strengths of this meta-analysis are its inclusion of a relatively large number of DM rodents and its focus on fucoidan’s effects on their blood glucose and lipid levels. Systematic assessments were carried out on various confounding factors and publication biases.

Nevertheless, this meta-analysis has several limitations.

(1) Our meta-analysis of blood glucose levels showed evidence of substantial heterogeneity; however, it could not be fully explained by the pre-specified parameters. The unexplained heterogeneity may potentially weaken the robustness of our results. To solve this problem, additional meta-analyses were conducted separately for the different types of DM rodent models, namely chemical- and diet-induced and genetically altered DM rodent groups. The results showed that fucoidan affected blood glucose levels only in diet-induced and genetically altered DM rodents; the sulfation rate of fucoidan and the family of brown algae were possible confounding factors for diet-induced and genetically altered DM rodents, respectively. Thus, additional research is needed to further clarify fucoidan’s role in diabetic hyperglycemia.

Moreover, in these animal studies, blinding the investigators to treatment (fucoidan vs. vehicle) was technically difficult.

(2) Some subgroups consisted of few studies. For instance, the family of brown algae was apparently a factor that influenced blood glucose levels of genetic DM rodents and TG levels of diet-induced DM rodents. However, fucoidans harvested from Fucaceae, Sargassaceae, etc., were used in only a few studies each; thus, the results have to be carefully re-assessed in the future before making a conclusion.

(3) The eligible studies mostly tested male rodents to reduce additional modifications to the results caused by the menstrual cycle or life stage. However, the outcomes could have been altered if more studies had included female rodents.

(4) The included studies used fucoidans that vary significantly in MW, degree of sulfation, algae source, and extraction methods. Because of the lack of standardization, the diversity of fucoidans could lead to high heterogeneity in the meta-analysis.

(5) The current study was conducted using preclinical data. Considering the physiological differences in glucose and lipid metabolism between humans and rodents, the results must be carefully assessed before they are translated to humans in clinical settings.

#### 4.2.2. Biological Properties of Fucoidan Against DM and Its Associated Complications

**Figure 10:**
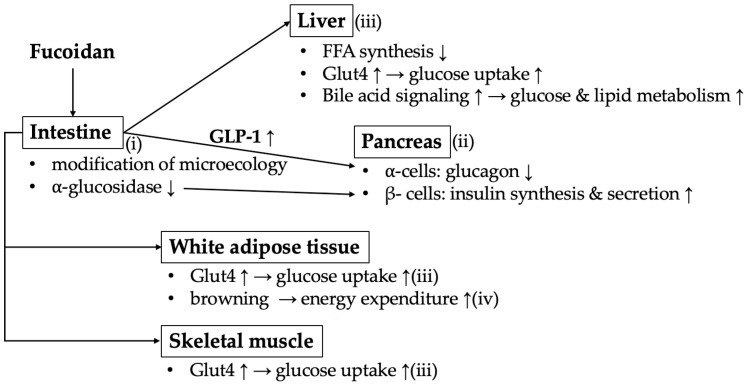
Possible biological mechanisms underlying fucoidan’s effect on glucose and lipid metabolism. Glut4: glucose transporter 4. FFA: free fatty acid. ↑: increase, ↓: decrease.

Finally, several biological mechanisms underlying fucoidan’s positive effect on glucose and lipid metabolism in DM rodents have been suggested (Figure 10) [3].

(i) Improvement of intestinal microecology:

Fucoidans were shown to restore expression of the intestinal epithelial tight junction proteins Zonula occludins-1 and Claudin-1 [17,39,69]. Thus, fucoidans might protect the small intestine from injury and restore its physiological barrier function. A suggested mechanism by which fucoidans repair the intestinal barrier function is inhibition of the activation of phosphatidylinositol 3-kinase (PI3K)/Akt/mechanistic target of rapamycin axis and lipo-polysaccharide (LPS)/toll-like receptor-4/nuclear factor-κB (NF-κB) signaling [30,32].

Several studies suggested a strong association between increased levels of *Firmicutes* (currently *Bacillota*) and *Proteobacteria* (currently *Pseudomonadota)* and reduced levels of *Bacteroidetes* (currently *Bacteroidetes*) and DM. Fucoidan administration increased microbiota diversity and richness by reducing *Proteobacteria* or modulating the *Firmicutes*-to-*Bacteroidetes* ratio in HFD-induced DM mouse models [17,26,39].

Moreover, fucoidans increase short-chain fatty acid (SCFA) levels in the colon by increasing SCFA-producing bacteria such as *Alloprevotella* [17,26,43]. SCFAs are fatty acids consisting of fewer than six carbon atoms and can stimulate G-protein-coupled receptor (GPR) 41, GRP43, and hydroxycarboxylic acid receptor 2 (GPR109a) [70,71,72]. SCFAs can reflect improvements in serum glucose and lipid levels in T2DM rodent models [73]. For instance, butyric acid promotes GPR43 and GPR109a signaling; propionic acid regulates energy homeostasis through both GPR41 and GPR43 pathways [17,71,72,74]. Through the activation of these receptors, SCFAs were shown to stimulate the secretion of gut hormones, including glucagon-like peptide-1 (GLP-1) and peptide YY (PYY), from the intestinal L-cells [75,76]. PYY_1–36_ is rapidly processed by dipeptidyl peptidase-4 to PYY_3–36_, which is highly selective for the Neuropeptide Y Y_2_-receptor, expressed in locations including the hypothalamus. Through receptor interaction, PYY exerts an inhibitory effect on appetite via the gut–brain axis [77]. Taken together, SCFAs indirectly suppress appetite, food intake, and weight gain, reducing the risk of T2DM.

(ii) Increased insulin synthesis and secretion in pancreas

An incretin hormone, GLP-1, is released from the intestine after meals [78]. GLP-1 stimulates pancreatic insulin secretion from β-cells via the GLP-1 receptor (GLP-1R) and suppresses glucagon release from α-cells. The GLP-1-mediated increase in cyclic adenosine monophosphate (cAMP) leads to activation of protein kinase A (PKA) and enhances signaling through exchange proteins directly activated by cAMP (Epac) [79,80,81]. PKA stimulates Ca^2+^ influx via L-type voltage-dependent calcium channels, and Epac enhances Ca^2+^ release from the endoplasmic reticulum [79,80,82,83]. Thus, both PKA and Epac promote insulin release from pancreatic β-cells. Moreover, GLP-1 increases insulin synthesis by activating the PKA–duodenal homeobox-1 (PDX-1) axis [81,84]. PDX-1 is a key transcription factor that promotes GLP-1R expression and insulin synthesis. Furthermore, GLP-1 prevents the loss of pancreatic β-cell mass under diabetic conditions; it also promotes the proliferation of β-cells and suppresses their apoptosis through mechanisms mediated by the PDX-1 and PI3K/Akt signaling pathways [47,85,86,87].

α-Glucosidase in the brush border of the small intestine acts as a carbohydrate hydrolase on the terminal bonds of starch and glycogen to release α-glucose. Fucoidan has been suggested to play a role in inhibiting α-galactosidase activity and improving pancreatic β-cell dysfunction and overall glucose metabolism [30].

(iii) Regulation of glucose and lipid metabolism in the liver

Glucose transporter 4 (Glut4), mainly expressed in striated muscles and adipose tissues, plays a critical role in glucose metabolism. Glut4 is translocated to the plasma membrane in an insulin-independent manner; it promotes *Glut4* transcription and improves glucose uptake and utilization in target cells [88]. The expression of *insulin receptor substrate-1* (*IRS-1*) and *Glut4* was reduced in HFD-induced DM mice; fucoidan administration in these mice might improve glucose uptake by restoring the levels of *IRS-1* and *Glut4* in the liver [17].

When insulin binds to its receptor, IRS-1/PI3K/Akt signaling is activated in the liver. Hepatic mRNA and protein expression of PI3K, Akt, and IRS were markedly reduced in DM mice, whereas administration of fucoidan restored hepatic PI3K/Akt signaling compared with vehicle [30].

Fucoidans inhibit FFA synthesis and promote FFA oxidation by down-regulating the FFA-synthesis-related genes *fatty acid synthase* (*Fas*), *leucine-rich repeat extensin* (*Lrx*), and *sterol regulatory element binding protein-1c* (*Srbp-1c*) and upregulating *peroxisome proliferator-activated receptor α* (*PPARα*) [17,32]. PPARα has been found to directly induce hepatic FFA oxidation [89].

Lastly, fucoidan regulates bile acid metabolism. Fucoidan treatment restored the secretion of secondary bile acids (taurolithocholic, nordeoxycholic, and ursodeoxycholic acids), which were reduced in DM conditions, and alleviated dyslipidemia through an intestinal flora–bile acid axis [19]. Bile acids are signaling molecules and bind to the receptors farnesoid X receptor (FXR) and Takeda G-protein-coupled receptor 5 (TGR5R) [90,91]. FXR is a nuclear receptor highly expressed in the intestine and liver; its activation suppresses lipogenesis and promotes FFA oxidation. TGR5 is another target that mediates interactions between bile acids and tissues/organs. Activation of intestinal TGR5 promotes GLP-1 secretion and improves glucose and lipid metabolism [92,93]. Fucoidan treatment was shown to restore FXR and TGR5 signaling in DM rodent models [19].

(iv) Browning white adipose tissue

Adipose tissue is classified into two categories, brown adipose tissue (BAT) and white adipose tissue (WAT). BAT consists of multi-compartmental adipocytes rich in mitochondria and is actively involved in thermogenesis [94]. Lin et al. showed that fucoidan can brown WAT in obese mice by upregulating expression of *uncoupling protein-1*, *PR domain containing 16*, *PGC-1α*, etc., which promote thermogenesis and energy expenditure [17].

In sum, fucoidan may play a crucial role in glucose and lipid metabolism by acting on various organs responsible for the onset and development of DM.

## 5. Conclusions

The current meta-analysis indicated that blood glucose and serum/plasma TG and TC levels were significantly reduced in fucoidan-treated DM rodents. Few clinical trials reported in English have been carried out to evaluate the effect of fucoidan on blood glucose and lipid levels in patients with DM or obesity/insulin resistance [8,9,10]. Our preclinical findings have limitations, as described above; however, the overall results are promising, and may help in promoting future preclinical and clinical research on fucoidan in people living with DM or obesity/insulin resistance.

## Figures and Tables

**Table 1:** Baseline characteristics of included studies.

Authors, Year	Fucoidan (MW)	Species of Brown Algae (Family)	Animal Models (Dose; mg/kgBW)	*n* (T/No-T)	Sex	Age or BW at the Baseline	Diet	Dose (mg/kg BW/Day)	Measured Data
Yu Y, 2025 [16]	① Fucoidan (345.6 kDa) ② LMWF (2.6 kDa)	*Saccharina japonica* (Laminariaceae)	Mice	5/5	m	6 wks	HFD	50	BG, TG, TC, HDL-C
Lin Q, 2025 [17]	Fucoidan (243.7 kDa)	*Saccharina japonica* (Laminariaceae)	Mice	6/6	m	6 wks	HFD	300	BG, TG, TC, HDL-C
Xue T, 2025 [18]	LMWF (8.84 kDa)	*Saccharina japonica* (Laminariaceae)	Rats	10/10	m	200 ± 20 g	HFSD	100	BG, TG, TC, HDL-C
Zhang B, 2024 [19]	LMWF (14.6 kDa)	*Saccharina japonica* (Laminariaceae)	Rats, STZ (ND)	8/8	m	200 ± 20 g	HFSD	100	BG, TG, TC, HDL-C
Chen L, 2024 [20]	Fucoidan (260 kDa)	*Undaria pinnatifida* (Alariaceae)	Mice	15/15	m	8 wks	HFD	200	BG
Zhong Z, 2024 [21]	Fucoidan (220–260 kDa)	*Undaria pinnatifida* (Alariaceae)	Mice	15/15	m	8 wks	HFD	200	BG
Gao H, 2024 [22]	Fucoidan [Sigma-Aldrich Co. LLC, St. Louis, MO, USA]	ND	NOD mice	10/10	f	6 wks	Control	600	BG
Puhari SSM, 2023 [23]	Fucoidan extracts	*Sargassum wightii* (Sargassaceae)	Rats, alloxan (150)	6/6	m	180–250 g	Control	300	BG, TG, TC, HDL-C
Lin Z, 2023 [24]	Fucoidan extracts	*Sargassum wightii* (Sargassaceae)	db/db mice	6/6	m	7–8 wks	Control	200	BG, TG, TC, HDL-C
He S, 2023 [25]	Fucoidan extracts	ND	Rats, STZ (35)	8/8	m	130 ± 10 g	HFD	70	BG, TG, TC, HDL-C
Zhang C, 2022 [26]	Fucoidan (89 kDa)	*Saccharina japonica* (Laminariaceae)	Mice, STZ (70)	8/8	m	6 wks	HFD	500	BG, TG, TC, HDL-C
Li Z, 2022 [27]	LMWF (8.1 kDa)	*Saccharina japonica* (Laminariaceae)	db/db mice	6/6	m	5 wks	Control	80	BG
Zhang X, 2022 [28]	Fucoidan (627.5 kDa)	*Saccharina japonica* (Laminariaceae)	Mice	7/7	m	4 wks	HFD	300	BG, TG, TC, HDL-C
Wang M-Z, 2022 [29]	Fucoidan (242.2 kDa) [Jilin Province Huinan Chonglong Bio-Pharmacy Co. Ltd., Jilin, China]	ND	Rats, STZ (35)	5/5	m	200–220 g	HFD	120	BG
Liu Y, 2022(1) [30]	Fucoidan (288.3 kDa)	ND	Rats, STZ (35)	8/8	m	80 ± 20 g	HFD	200	BG, TG, TC, HDL-C
Liu Y, 2022(2) [31]	Fucoidan (288.3 kDa)	ND	Rats, STZ (35)	8/8	m	4 wks	HFD	200	BG
Deng Z, 2021 [32]	LMWF (7.2 kDa)	*Saccharina japonica* (Laminariaceae)	Mice	10/10	m	4 wks	HFD	200	BG, TG, TC, HDL-C
Wang Y, 2021 [33]	LMWF (8.84 kDa)	*Saccharina japonica* (Laminariaceae)	Rats, STZ (ND)	10/10	m	200 ± 20 g	HFSD	200	BG, TG, TC
Zhang Y, 2021 [34]	Fucoidan (SFF) (ND)	*Sargassum fusiforme* (Sargassaceae)	Mice	8/8	m	7 wks	HFD	200	BG, TG, TC, HDL-C
Wu Q, 2021 [35]	Fucoidan (205.8 kDa)	*Sargassum fusiforme* (Sargassaceae)	Mice, STZ (40)	8/8	m	6 wks	HFD	100	BG, TG, TC, HDL-C
Wang L, 2021 [36]	Fucoidan [Sigma-Aldrich Co. LLC]	*Fucus vesiculosus* (Fucaceae)	Mice	8/8	m	8 wks	HFD	10	BG, TG, TC
Kizilay G, 2021 [37]	Fucoidan (ND)	ND	Rats, STZ (60)	9/9	m	3 mos	Control	100	BG
Xu J, 2021 [38]	LMWF (9.554 kDa)	*Saccharina japonica* (Laminariaceae)	Rats, STZ (ND)	14/10	m	240–250 g	HFSD	200	BG
Zhang Y, 2020 [39]	Fucoidan (ND)	*Sargassum fusiforme* (Sargassaceae)	Mice	10/10	m	32 ± 2 g	HFD	200	BG, TG, TC, HDL-C
Peng Y, 2020 [40]	Fucoidan extracts	*Kjellmaniella crassifolia* (Laminariaceae)	Rats, STZ (ND)	8/8	m	180 ± 10 g	HFD	150	BG, TG, TC, HDL-C
Aleissa MS, 2020 [41]	Fucoidan capsule [Absonutrix Lyfetrition, Colfax, NC, USA]	*Saccharina japonica* (Laminariaceae)	Rats, STZ (50)	8/8	m	180–200 g	Control	100	BG, TC
Wang X, 2019 [42]	Fucoidan (ND)	*Fucus vesiculosus* (Fucaceae)	Mice, STZ (150)	12/12	m	8 wks	HFD	100	BG, TG, TC, HDL-C
Cheng Y, 2019 [43]	Fucoidan (205.8 kDa)	*Sargassum fusiforme* (Sargassaceae)	Mice, STZ (40)	10/10	m	20 ± 2 g	Control	100	BG
Liu T, 2018 [44]	LMWF (~7 kDa)	*Saccharina japonica* (Laminariaceae)	db/db mice	10/14	ND	10–12 wks	Control	60	BG, TG, TC, HDL-C
Zheng Y, 2018 [45]	LMWF (6.5 kDa)	*Saccharina japonica* (Laminariaceae)	db/db mice	8/8	m	10 wks	Control	80	BG, TG, TC
Wang Z, 2018 [46]	LMWF (6.5 kDa)	*Saccharina japonica* (Laminariaceae)	GK rats	6/6	m	20 wks	Control	40	BG, TG, TC, HDL-C
Yu W-C, 2017 [47]	LMWF (0.8 kDa)	*Sargassum hemiphyllum* (Sargassaceae)	Mice, STZ (75)	8/8	m	6 wks	Control	300	BG
Lin H-TV, 2017 [48]	LMWF (0.8 kDa)	*Sargassum hemiphyllum* (Sargassaceae)	db/db mice	8/8	m	9 wks	Control	300	BG
Yang X-D, 2017 [49]	Fucoidan [Sigma-Aldrich Co. LLC]	ND	Rats, STZ (60)	10/10	m	180–200 g	Control	100	BG
Liang Z, 2016 [50]	LMWF (6.5 kDa)	*Saccharina japonica* (Laminariaceae)	Rats, STZ (65)	8/8	m	180–200 g	Control	100	BG, TG, TC, HDL-C
Shan X, 2016 [51]	Fucoidan (ND)	*Fucus vesiculosus* (Fucaceae)	db/db mice	12/12	m	5–7 wks	Control	20	BG
Heeba GH, 2015 [52]	Fucoidan [Sigma-Aldrich Co. LLC]	*Fucus vesiculosus* (Fucaceae)	Rats	7/7	m	180–200 g	HFD	43	BG, TG, TC
Jiang X, 2015 [53]	Fucoidan [Sigma-Aldrich Co. LLC]	ND	GK rats	8/8	m	6 wks	Control	75	BG
Wang Y, 2015 [54]	Fucoidan [Sigma-Aldrich Co. LLC]	*Fucus vesiculosus* (Fucaceae)	GK rats	8/8	m	6 wks	Control	75	BG
Wang J, 2014 [55]	Fucoidan (87 kDa)	*Saccharina japonica* (Laminariaceae)	Rats, STZ (50)	12/12	m	ND	Control	300	BG
Cui W, 2014 [56]	LMWF (7 kDa)	*Saccharina japonica* (Laminariaceae)	GK rats	8/15	m	12 wks	Control	200	BG, TG, TC, HDL-C
Yang W, 2013 [57]	LMWF (~7 kDa)	*Saccharina japonica* (Laminariaceae)	Mice, STZ (60)	18/16	m	6–8 wks	Control	200	BG
Jeong Y-T, 2013 [58]	LMWF (1 kDa)	*Undaria pinnatifida* (Alariaceae)	db/db mice	6/6	m	8 wks	Control	500	BG, TG, TC, HDL-C
Wang J, 2013 [59]	Fucoidan (ND)	*Saccharina japonica* (Laminariaceae)	Rats, alloxan (50)	10/10	ND	180–220 g	Control	600	BG, TG, TC, HDL-C
Kim K-J, 2012 [60]	① Fucoidan extracts ② LMWF (<5 kDa)	*Undaria pinnatifida* (Alariaceae)	db/db mice	8/8	f	ND	Control	45	BG

*n*: the number of animals; T/No-T: treated by fucoidan/vehicle; ND: not described; HFD: high-fat diet; HFSD: high-fat high-sugar diet; BG: blood glucose.

**Table 2:** Results of subgroup analysis for blood glucose levels of genetically altered DM rodent models.

TG Levels							
Subgroups	Effect Size					Heterogeneity (*I*^2^)	Test of Group Difference (*p*)
	No. of Studies	g	95% CI		*p*-Value		
**Rodent type**							
mice	10	−1.56	−2.16	−0.96	<0.001	66.71	0.93
rats	4	−1.48	−3.15	0.19	0.018	88.51	
**MW of fucoidan**							
HMWF	6	−1.98	−2.67	−1.28	0.074	50.01	0.43
LMWF	7	−1.18	−2.30	−0.06	<0.001	87.43	
not described	1	−2.08	−3.05	−1.11	–	–	
**Family of brown algae**							
Alariaceae	3	−3.13	−5.01	−1.24	0.040	78.01	<0.001 **
Fucaceae	2	−2.47	−3.47	−1.47	0.230	30.51	
Laminariaceae	5	−0.41	−0.89	0.06	0.232	21.71	
Sargassaceae	2	−1.35	−2.13	−0.57	0.855	0.00	
not described	2	−1.89	−3.68	−0.09	0.027	79.49	

*p*: *p*-value, ** *p* < 0.01.

**Table 3:** Results of subgroup analysis for serum/plasma TG and TC levels of DM rodent models.

**TG Levels**							
**Subgroups**	**Effect Size**					**Heterogeneity (*I*^2^)**	**Test of Group Difference (*p*)**
	**No. of Studies**	**g**	**95% CI**		***p*-Value**		
**DM rodent models**							
chemical	10	−3.14	−4.70	−1.57	<0.001	92.84	0.004 **
diet	10	−2.63	−3.46	−1.80	0.001	72.61	
genetic	6	−1.15	−1.77	−0.54	0.068	51.90	
**Rodent type**							
mice	15	−1.92	−2.93	−1.45	0.003	57.40	0.13
rats	11	−2.29	−4.21	−1.62	<0.001	93.71	
**MW of fucoidan**							
HMWF	12	−2.67	−3.61	−1.74	<0.001	81.79	0.13
LMWF	10	−2.70	−4.01	−1.40	<0.001	90.44	
not described	4	−1.24	−2.42	−0.05	<0.001	83.28	
**Family of brown algae**							
Alariaceae	1	−1.33	−2.50	−0.16	–	–	0.33
Fucaceae	3	−1.65	−2.25	−1.05	0.522	0.00	
Laminariaceae	15	−2.66	−3.62	−1.70	<0.001	87.76	
Sargassaceae	5	−1.98	−3.18	−0.79	0.006	79.22	
not described	2	−4.72	−11.85	2.41	<0.001	94.80	
**TC Levels**							
**Subgroups**	**Effect Size**					**Heterogeneity (*I*^2^)**	**Test of Group Difference (*p*)**
	**No. of Studies**	**g**	**95% CI**		***p*** **-Value**		
**DM rodent models**							
chemical	11	−3.57	−5.56	−1.59	<0.001	95.82	0.02 *
diet	10	−2.12	−2.98	−1.26	<0.001	76.14	
genetic	6	−1.07	−1.64	−0.50	0.116	44.38	
**Rodent type**							
mice	15	−1.50	−1.91	−1.09	<0.001	48.03	0.03 *
rats	12	−3.59	−5.46	−1.72	<0.001	95.46	
**MW of fucoidan**							
HMWF	13	−4.04	−6.38	−1.70	<0.001	97.11	0.04 *
LMWF	10	−2.19	−3.17	−1.22	<0.001	84.73	
not described	4	−0.82	−2.03	0.38	<0.001	85.07	
**Faily of brown algae**							
Alariaceae	1	−2.15	−3.50	−0.82	–	–	0.58
Fucaceae	3	−1.58	−2.35	−0.82	0.205	38.49	
Laminariaceae	16	−2.41	−3.35	−1.48	<0.001	88.16	
Sargassaceae	5	−2.70	−6.44	1.04	<0.001	98.06	
not described	2	−5.65	−13.69	2.38	<0.001	94.62	

*p*: *p*-value, * *p* < 0.05, ** *p* < 0.01.

**Table 4:** Subgroup analyses of serum/plasma TG levels in chemical-induced DM rodent models.

TG Levels							
Subgroups	Effect Size					Heterogeneity (*I*^2^)	Test of Group Difference (*p*)
	No. of Studies	g	95% CI		*p*-Value		
**Rodent type**							
mice	3	−2.06	−3.82	−0.30	0.004	86.21	0.26
rats	7	−3.67	−5.83	−1.51	<0.001	93.26	
**MW of fucoidan**							
HMWF	6	−3.52	−5.62	−1.41	<0.001	91.86	0.03 *
LMWF	2	−4.71	−6.99	−2.42	0.097	63.68	
not described	2	−0.69	−2.78	1.41	0.001	91.10	
**Family of brown algae**							
Fucaceae	1	−1.76	−2.68	−0.84	-	-	0.55
Laminariaceae	5	−3.10	−5.21	−1.06	<0.001	90.38	
Sargassaceae	2	−2.84	−7.14	1.47	0.001	91.54	
not described	2	−4.72	−11.85	2.41	<0.001	94.80	

*p*: *p*-value, * *p* < 0.05.

**Table 5:** Subgroup analysis for serum/plasma TG levels of diet-mediated DM rodent models.

Subgroups	Effect Size					Heterogeneity (*I*^2^)	Test of Group Difference (*p*)
	No. of Studies	g	95% CI		*p*-Value		
**Rodent type**							
mice	8	−2.24	−2.86	−1.63	0.09	42.85	0.37
rats	2	−4.40	−9.10	0.30	<0.001	92.26	
**MW of fucoidan**							
HMWF	5	−2.20	−2.94	−1.45	0.211	35.37	0.23
LMWF	3	−4.30	−6.90	−1.70	0.002	83.13	
not described	2	−1.81	−2.97	−0.66	0.128	56.77	
**Family of brown algae**							
Fucaceae	2	−1.58	−2.44	−0.71	0.275	16.00	0.04 *
Laminariaceae	6	−3.41	−4.60	−2.23	0.017	68.00	
Sargassaceae	2	−1.81	−2.97	−0.66	0.128	56.77	

*p*: *p*-value, * *p* < 0.05.

**Table 6:** Summary effects of fucoidan administration on the blood glucose and serum/plasma TG, TC, HDL-C levels of the three types of DM rodent models.

DM Models	BG	TG	TC	HDL-C
Chemical-induced	No effect	Reduced * (treatment period)	Reduced *	No effect
Diet-induced	Reduced * (sulfation rate of fucoidan)	Reduced * (sulfation rate of fucoidan)	No effect	
Genetic	Reduced * (family of brown algae)	Reduced	Reduced	

* High heterogeneity (*I*^2^ > 75%) is indicated. Identified covariates are described in parentheses. BG: blood glucose.

**Table 7:** GRADE assessment of the quality of evidence.

Outcomes	№ of Rodents (Studies)	Certainty of the Evidence (GRADE)	Impact
Blood glucose levels follow-up: range 4 weeks to 24 weeks	833 (47 RCTs)	⨁◯◯◯ Very low	Very high heterogeneity. Publication bias. No significant cofounding factor was identified in this analysis.
Blood glucose levels in diet-induced DM rodents follow-up: range 4 weeks to 24 weeks	212 (12 RCTs)	⨁⨁◯◯ Low	Very high heterogeneity. The sulfation rate of fucoidan was identified as a covariate.
Blood glucose levels in genetic DM rodents follow-up: range 4 weeks to 24 weeks	235 (14 RCTs)	⨁⨁⨁◯ Moderate	Very high heterogeneity. The family of brown algae was identified as a significant confounding factor.
TG levels follow-up: range 4 weeks to 24 weeks	415 (26 RCTs)	⨁⨁⨁◯ Moderate	Very high heterogeneity. Type of DM models and the treatment period were found to be significant confounding factors.
TC levels follow-up: range 2 weeks to 16 weeks	431 (27 RCTs)	⨁⨁⨁◯ Moderate	Very high heterogeneity. Types of DM models and rodents and the MW of fucoidan were detected as significant confounding factors.

## Data Availability

The original contributions presented in the study are included in the article/Supplementary Material, further inquiries can be directed to the corresponding author.

## Supplementary Materials

The following supporting information can be downloaded at https://www.mdpi.com/article/10.3390/nu18071155/s1. Table S1: PRISMA checklist; Table S2: Risk of bias; Table S3: Subgroup analyses of blood glucose levels in DM rodents.
